# Immunological Events, Emerging Pharmaceutical Treatments and Therapeutic Potential of Balneotherapy on Osteoarthritis

**DOI:** 10.3389/fphar.2021.681871

**Published:** 2021-06-30

**Authors:** Anna Scanu, Lucrezia Tognolo, Maria Chiara Maccarone, Stefano Masiero

**Affiliations:** ^1^ Rheumatology Unit, Department of Medicine-DIMED, University of Padua, Padua, Italy; ^2^ Department of Neurosciences, Physical Medicine and Rehabilitation School, University of Padua, Padua, Italy

**Keywords:** immune system, osteoarthritis, balneotherapy, inflammation, disease-modifying OA drug

## Introduction

Although hypotheses have been proposed, the exact pathophysiological mechanisms of osteoarthritis (OA) still remain unknown. Evidence suggests that immunological events, immune-neuroendocrine dysregulation and the presence of low-grade local and systemic inflammation play a key role in the pathogenesis and progression of this disease (Scanzello et al., 2015; Galvez et al., 2017; Chow et al., 2020; Woodell-May et al., 2020). Traditional therapies for OA focus on minimizing the symptoms, but not cure the arthritis. If none of these measures are effective, surgery is the next option. However, any medical or surgical treatment can have severe side effects. Balneotherapy is a common practice for the treatment and rehabilitation of OA patients whose role in modern medicine needs to be better defined. Studies have demonstrated that the beneficial effects of balneotherapy are mediated by regulation of inflammatory cells and mediators (Gálvez et al., 2018). This article aims to provide a standpoint on the possible involvement of immune system in these processes, and why it should be considered a target for therapy in such instances, based on published literature. Furthermore, we propose that the balneotherapy effectiveness in this context be better examined in future studies, in order to expand its employment alone or as a complement to other treatments in the OA management.

## Immunological Events in OA

It is increasingly recognized that immune cells and their molecular mediators play a part in OA development. Enhanced leukocyte infiltration in the synovium and the presence of activate macrophages (M1) in synovial fluid (SF) have been identified in OA patients (Deligne et al., 2015; Kraus et al., 2016; Liu et al., 2018). Concomitantly, pro-inflammatory cytokines are produced locally by infiltrating and resident cells in early and end-stage of OA, independently or on collaboration with other mediators (Goldring et al., 2011; Punzi et al., 2016).

The main triggering of these events seems to be the activation of innate immunity by damage-associated molecular patterns, including extracellular matrix fragments, high mobility box 1, uric acid, complement system, S100 proteins, and heat shock proteins (HSPs) that are released into the joint after trauma or age-related processes (Gobezie et al., 2007; Scanzello et al., 2008; Ke et al., 2015). These molecules, generated in part by oxidative stress, are able to bind in synovial cells pathogen-recognition receptors, such as toll-like receptors, the receptor for advanced glycation end products and the NLRP3 inflammasome, and induce pro-inflammatory mediators production (Kim et al., 2006; Steenvoorden et al., 2006; McAllister et al., 2018). Indeed, the levels of several inflammatory cytokines, such as IL-1β and IL-6, are higher in serum from OA compared to healthy subjects (Sohn et al., 2012); whereas elevated IL-6, IL-8 and CCL2 were found in OA SF (Kaneko et al., 2000; Li et al., 2015; Oliviero et al., 2020). Increased IL-1β, IL-6, TNF-α and IL-8 concentrations are also detected in synovial tissues and articular cartilage of OA patients (Ma et al., 2015; Ni et al., 2015; Böhm et al., 2016).

Moreover, high concentrations of cytokines could be secreted from senescent cells, which accumulate in the synovium and in cartilage surface, thus predisposing the joint to OA development (Jeon et al., 2017).

In turn, these inflammatory factors, carried by the SF, can activate chondrocytes to produce metalloproteinases (MMPs) which result in further cartilage damage (Nefla et al., 2016). The activation of NF-kB, PI3K/AKT/mTOR, and Wnt/β-Catenin signaling pathways seems to play a key role in these processes (Rigoglou et al., 2013; Zhou et al., 2017; Sun et al., 2020).

Concomitantly, the upregulation of other factors, such as inducible nitric oxide synthase, nitric oxide (NO), cyclooxygenase-2, prostaglandin E2 (PGE2), A Disintegrin And Metalloproteinase with Thrombospondin motif (ADAMTS)-5, ADAMTS-4, VEGF, TGF-β and Nerve Growth factor (NGF) exert their effect influencing the OA progression (Chow et al., 2020).

Finally, microRNA can be involved in OA processes by activating different signaling pathways, and thus promoting inflammatory factor release (Wu et al., 2019).

## Current and Future Pharmaceutical Therapy for OA

Current pharmacological treatments for OA are focused on relieving symptoms and improving functional status. Paracetamol and nonsteroidal anti-inflammatory drugs (NSAIDs) are the first line medication choices for pain management, but their long-term use is associated with side effects.

Other common options include intra-articular corticosteroid or hyaluronic acid (HA) injections. Both treatments are effective at reducing pain in OA patients. However, intra-articular corticosteroid injections have shown a short duration of action, resulting in the need for repeated administration, which can lead to local and systemic side effects (Raynauld et al., 2003). The durability of pain reduction has been demonstrated longer after intra-articular HA injection when compared to corticosteroids (Colen et al., 2010). Nevertheless, due to the heterogeneity of approach, further studies should be conducted to confirm this hypothesis.

**FIGURE 1:**
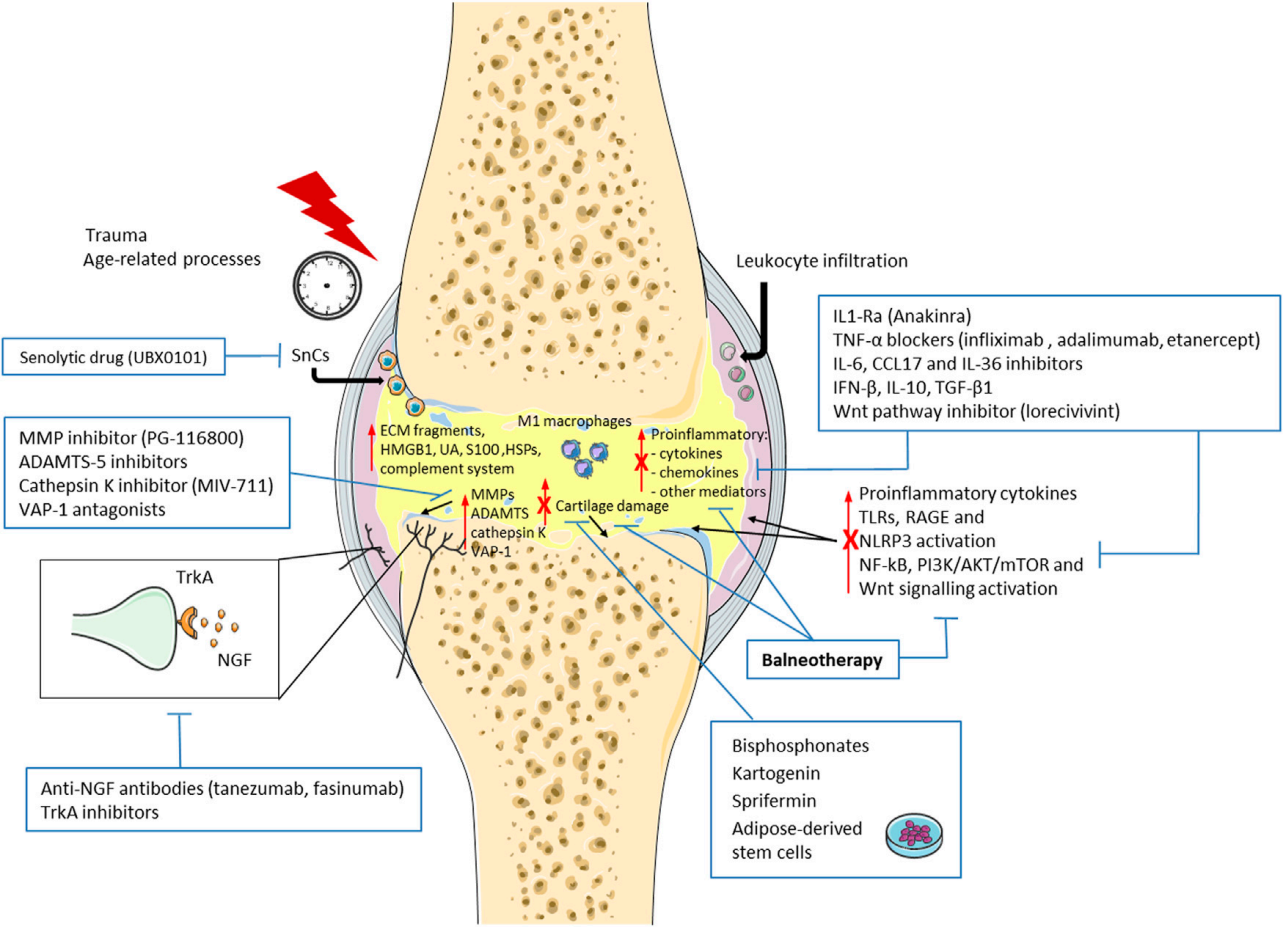
Schematic representation of the new emerging therapies targeting immune system and immunomodulatory properties of balneotherapy in osteoarthritis (OA). Immunological events and low-grade inflammation play a key role in the pathogenesis and progression of OA. Damage-associated molecular patterns (DAMPs), including extracellular matrix (ECM) fragments, high mobility box 1 (HMGB1), uric acid (UA), complement system, S100 proteins, and heat shock proteins (HSPs) are released into the joint after trauma or age-related processes. These molecules, bind Toll-like receptors (TLRs), the receptor for advanced glycation end products (RAGE) and the intracellular NLRP3 inflammasome, and induce the production of pro-inflammatory mediators, such as cytokines and chemokines. Concomitantly, leukocyte infiltration, presence of activate macrophages (M1) and senescent cells (SnCs), levels metalloproteinases (MMPs), A Disintegrin And Metalloproteinase with Thrombospondin motif (ADAMTS), vascular adhesion protein-1 (VAP-1) and Nerve Growth factor (NGF) are enhanced, thus promoting cartilage damage and pain. Finally, NF-kB, PI3K/AKT/mTOR, and Wnt/β-Catenin signaling pathways are activated in these processes. New emerging treatment and balneotherapy have demonstrated a decrease in OA progression through immunomodulatory properties. **↑** increase; **T** inhibition; **X** reduction by balneotherapy.

Therefore, new treatments are required to prevent OA structural changes and progression. Molecules involved in the OA pathophysiological processes, especially in immunological events, could be an interesting candidate as therapeutic target. In this context, several drugs have demonstrated disease-modifying OA effects in preclinical and clinical studies (Figure 1).

Cytokine inhibitors represent a putative class of these agents. As IL-1β is thought to play a key role in OA development, and intra-articular injection of IL-1 receptor antagonist (IL1-Ra) has demonstrated to improve KOOS (Knee Injury and Osteoarthritis Outcome Score) in patients with anterior cruciate ligament (ACL) tear (Krauss et al., 2012), particular attention has focused on this cytokine. However, a randomized, controlled study evaluating the clinical response, safety, and tolerability of a single intra-articular injection of IL-1Ra in patients with knee OA, revealed no improvements in symptoms when compared with placebo (Chevalier et al., 2009). Results from a phase I trial investigating the adenovirus-mediated IL-1Ra gene transfer in knee OA are pending (Latourte et al., 2020).

TNF inhibition has also been investigated using IgG monoclonal antibodies (infliximab or adalimumab) or circulating receptor fusion protein (etanercept). Treatment with anti-TNF-α blockers has demonstrated a decrease in disease progression but not in symptoms in patients with hand OA (Verbruggen et al., 2012; Kloppenburg et al., 2018; Loef et al., 2018).

Other ongoing studies are evaluating inhibition of pro-inflammatory cytokines (IL-6, CCL17 and IL-36), or intra-articular effects of anti-inflammatory cytokines (IFN-β and IL-10) (Latourte et al., 2020).

Intra-articular release of TGF-β1 by retrovirally transduced human chondrocytes has also demonstrated good results, with improvement in cartilage damage and symptoms in patients with knee OA (Ha et al., 2012; Guermazi et al., 2017; Kim et al., 2018; Lee et al., 2019).

Matrix-degrading enzyme inhibition may be another attractive approach to attenuate cartilage damage, even though musculoskeletal toxicity has been observed after PG-116800 administration to patients with knee OA (Krzeski et al., 2007). Promising results may be obtained through aggrecanase inhibition. Indeed, ADAMTS-5 small molecules inhibitors or neutralizing antibodies have shown protective effects on cartilage and safety profile *in vivo* and in clinical studies (Malfait et al., 2019).

Similar effects were observed after intra-articular treatment with UBX0101, a senolytic drug capable of removing senescent cells accumulated in the joint (Jeon et al., 2017). In addition, less cartilage loss accompanied by bone remodeling reduction was found after administration of MIV-711, a cathepsin K inhibitor (Lindstrom et al., 2018; Conaghan et al., 2019). Analogous could be confirmed for bisphosphonates in early OA (Lane, 2018).

A chondroprotective activity, with signs of cartilage repair after injury has been also reported after intra-articular injection of adipose-derived stem cells in experimental OA (ter Huurne et al., 2012), but results obtained from clinical trials were not convincing (Pers et al., 2016; Emadedin et al., 2018; Freitag et al., 2019; Kim et al., 2019; Lee et al., 2019). Evaluations on emerging drugs promoting chondrogenesis, such as kartogenin and sprifermin, are ongoing (Eckstein et al., 2020; Johnson et al., 2020).

Studies on new therapies targeting signaling have identified a small-molecule Wnt pathway inhibitor, lorecivivint, as a potential disease-modifying OA drug (DMOAD). Administration of this agent facilitated cartilage regeneration in a rodent acute OA model (Deshmukh et al., 2018), and improved pain and function, with good safety and tolerability, in subjects with unilateral symptomatic knee OA (Yazici et al., 2017; Yazici et al., 2020).

Other interesting strategies to reduce pain have been observed using anti-NGF antibodies, such as tanezumab and fasinumab, even though increased risk of rapidly progressive OA was observed after patient treatment (Lane et al., 2010; Hochberg et al., 2016; Dakin et al., 2019; Berenbaum et al., 2020). The use of high-affinity NGF receptor (TrkA) inhibitors could be a viable alternative to avoid side effects (Krupka et al., 2019). New treatments targeting pain include inhibition of vascular adhesion protein-1 (VAP-1), an amine oxidase that increases in OA cartilage. The results of a Phase II clinical trial on a VAP-1 antagonist have not yet been published (Vakal et al., 2020).

Finally, platelet-rich plasma has been recently considered as innate immune response modulator, even though administration protocols and OA phenotypes target have to be refined (Andia et al., 2021).

Despite a number of potential DMOAD molecules have been identified, currently there are no approved drugs, and further studies are needed in this area.

## Balneotherapy On OA

Besides pharmacological treatment, non-pharmacological interventions play a significant role in the OA prevention and treatment. Balneotherapy is one of the most common non-pharmacological approach for musculoskeletal complaints and rheumatic diseases that has demonstrated benefit for disease symptoms, and has been recently recommended by OARSI guidelines as a strategy for patients with multi-joint OA and comorbidities (Masiero, 2008; McAlindon et al., 2014; Cozzi et al., 2018; Masiero et al., 2018). Although the biological mechanisms underlying balneotherapy are not completely understood, it has been reported that it exerts some beneficial effects on the immune system due likely to its chemical, thermal and mechanical properties (Fioravanti et al., 2011; Tenti et al., 2015) (Figure 1).

*In vitro* researches have highlighted the immunomodulatory properties of mineral waters using animal or human OA cell cultures, or experimental models able to reproduce OA conditions in articular tissues. Treatment with H2S donors have showed anti-inflammatory and anti-oxidant activities limiting the MAPK/ERK and NF-kB pathway activation and reducing the production of several factors implicated in OA, including IL-6, IL-8, NO, PGE2 and MMP-13 (Kloesch et al., 2011; Burguera et al., 2014; Cheleschi et al., 2020). However, the applicability of cell culture models in this context remains a subject of debate since, under non-experimental conditions, the joint cells are never in direct contact with thermal water. The use of animal models may be more appropriate to identify the mechanisms involved, but so far only four studies have been conducted. In experimental OA murine models, balneotherapy decreased the levels of pro-inflammatory mediators in articular tissues, and systemic IL-1β and NO (Caraglia et al., 2005; Tékus et al., 2018; Kim et al., 2020; Vaamonde-García et al., 2020).

Also the number of studies in patients is limited: currently only three active projects are listed in clinical trial registry (ClinicalTrials.gov, 2020). However these confirm preclinical evaluations. Indeed, decreased serum levels of pro-inflammatory mediators (PGE2, leukotriene B4, IL-1β, TNF-α, IL-8, IL-6, and eHsp72) and expression of microRNA related to cartilage degradation were observed after mud pack treatments in OA patients (Bellometti et al., 1998; Giannitti et al., 2017; Ortega et al., 2017; Maccarone et al., 2020). Interestingly, balneotherapy increased circulating cortisol concentration that enhanced monocyte chemotaxis to damaged tissue and their switch to an anti-inflammatory phenotype (Galvez et al., 2020).

Of note, the association of mud-bath and glucosamine sulfate or ultrasound therapy has indicated improvement in pain, function and life quality in OA patients; thus suggesting combined treatment as an important therapeutic approach (Peluso et al., 2016; Király et al., 2021).

## Conclusion

On these bases, we believe that therapeutic intervention to modulate immunological events could be an innovative strategy to contain OA progression, and balneotherapy may represent an interesting candidate to support pharmacological therapy. Strategies to improve research in this field need yet to be further refined and implemented. In this context, to identify the molecules and mechanisms associated with this pathological condition and spa treatment, alone or combined with other therapies, with *in vivo* and clinical studies, is crucial to validate the effectiveness and importance of pharmacological and non-pharmacological approaches.
